# Evaluation of Ginsenosides and Their Derivatives From *Panax ginseng* as Aromatase Inhibitors for Breast Cancer Treatment—An *in silico* study

**DOI:** 10.1155/bmri/5538317

**Published:** 2026-02-16

**Authors:** Jayasri Gokila Madhan, Surya Sekaran, Rajeswari Nambirajan Akshaya, Khavyanjali Venkatesan, Kavin Kirupanandasamy, Kiruthika Vijayakumar, Nancy Jenifer, Benedict Christopher Paul

**Affiliations:** ^1^ Department of Biotechnology, Faculty of Biomedical Sciences and Technology, Sri Ramachandra Institute of Higher Education and Research, Chennai, India, sriramachandra.edu.in

**Keywords:** aromatase inhibitors, breast cancer, cytochrome P450, ginsenosides, in silico, *Panax ginseng*, public health

## Abstract

Breast cancer, particularly estrogen receptor‐positive subtypes, is a leading cause of cancer‐related mortality worldwide. Aromatase inhibitors, which target estrogen biosynthesis, are a cornerstone of therapeutic intervention. *Panax ginseng*, a widely recognized medicinal herb, contains bioactive compounds known as ginsenosides, which possess various pharmacological activities, including anticancer properties. This study is aimed at evaluating the potential of ginsenosides and their derivatives from *Panax ginseng* as aromatase inhibitors for breast cancer treatment through *in silico* methods. Molecular docking studies were conducted to investigate the binding affinities of ginsenosides to aromatase, a critical enzyme in estrogen biosynthesis. The results indicated that several ginsenosides exhibited strong binding affinities, with Protopanaxadiol demonstrating the highest affinity (−10.0 kcal/mol) and an estimated *K* *i* value of 46.77 nM. Molecular dynamics simulations confirmed the stability of the protopanaxadiol‐aromatase complex, highlighting its consistent binding interactions and minimal structural fluctuations. Pharmacokinetic evaluations suggested that most ginsenosides adhered to drug‐likeness criteria, although certain derivatives showed deviations that may affect bioavailability. However, toxicity predictions revealed a predominantly low toxicity profile, with some concerns related to hepatotoxicity and mutagenicity for specific compounds. The findings from this study highlight the potential of ginsenosides and their derivatives as viable candidates and protopanaxadiol as a promising lead compound for further experimental investigation as aromatase inhibitors, offering a promising alternative or adjunct to conventional breast cancer therapies.

## 1. Introduction

Breast cancer is a leading cause of cancer‐related mortality in women, underscoring the urgent need for novel therapeutic strategies. It has surpassed lung cancer as the most frequently diagnosed malignancy, accounting for 6.9% of cancer deaths worldwide [1]. Central to the progression of many breast cancers is the enzyme aromatase, encoded by the CYP19A1 gene, which catalyzes the conversion of androgens to estrogens in tissues such as the ovaries, adipose tissue, and the brain.

As a highly heterogeneous disease, breast cancer is classified into subtypes based on hormone receptor expression, including estrogen receptor‐positive (ER+), progesterone receptor‐positive (PR+), human epidermal growth factor receptor‐positive (HER2+), and triple‐negative (TNBC) [2]. Aromatase inhibitors are the standard of care for ER+ breast cancer, which is a significant global health concern, particularly in postmenopausal women [3].

The therapeutic rationale for targeting aromatase is strong, as breast cancer tissues express the enzyme at higher levels and produce more estrogen than nonmalignant cells [4]. Aromatase, a member of the cytochrome P450 (CYP) superfamily, catalyzes the rate‐limiting step in estrogen biosynthesis, making it a critical drug target. Although increased CYP19A1 expression has been linked to breast cancer, its direct correlation with risk and survival requires further elucidation [5]. The medicinal herb *Panax ginseng* is a source of bioactive compounds known as ginsenosides, which possess extensive pharmacological properties. In oncology, ginsenosides have been demonstrated to exert anticancer effects by inhibiting cell proliferation, inducing apoptosis and suppressing angiogenesis through the modulation of key signaling pathways. Specific ginsenosides, such as Rg3 and Rh2, have been shown to inhibit metastasis and enhance the efficacy of conventional chemotherapy drugs, highlighting their potential as complementary therapeutics [6, 7].

Crucially, the therapeutic potential of ginseng extends to the direct modulation of estrogen biosynthesis. Studies suggest that ginsenosides can inhibit aromatase activity, thereby reducing estrogen production and potentially slowing the growth of hormone‐dependent tumors. This positions ginseng‐derived compounds as potential natural aromatase inhibitors, offering a promising alternative to synthetic drugs ([8]).

Given the documented anticancer properties of ginsenosides and their putative role in aromatase inhibition, this study employs an in silico approach to investigate their molecular interactions with the aromatase enzyme. Molecular docking provides an efficient method to predict the binding affinities and modes of these compounds within the enzyme′s active site. This research, therefore, is aimed at evaluating ginsenosides and their derivatives as potential aromatase inhibitors, providing a computational basis for identifying novel, plant‐based therapeutic candidates for hormone‐dependent breast cancer that may offer fewer side effects than current synthetic options.

## 2. Materials and Methods

### 2.1. Ligand Choice and Preparation

The choice of ginsenosides′ and derivatives′ chemical structure was based upon their reported bioactivities as listed in available literature [9]. The selected compounds include within their scope protopanaxatriol, protopanaxadiol, oleanolic acid, ocotillol, 20(S)‐protopanaxadiol (R1 = Glc, R2 = H), 20(R)‐protopanaxadiol (R1 = H, R2 = O − Glc), Ginsenoside Rh4 (R1 = H, R2 = O − Glc), Ginsenoside Rk3 (R1 = H, R2 = O − Glc), 20(S)‐protopanaxatriol (R1 = Glc, R2 = H), and 20(S)‐protopanaxatriol (R1 = H, R2 = Glc).

**Figure 1:**
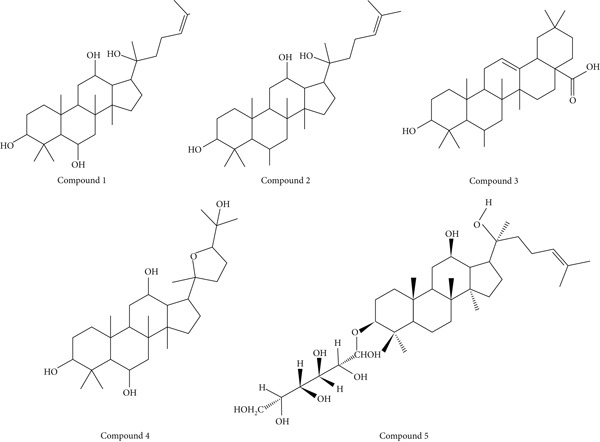
Chemical structures of ginsenosides and their derivatives selected for analysis in this study—(1) protopanaxatriol, (2) protopanaxadiol, (3) oleanolic acid, (4) ocotillol, (5) 20(S)‐protopanaxadiol (R1 = Glc, R2 = H), as reported in the literature [9]. All structures were drawn using ChemDraw software.

**Figure 2:**
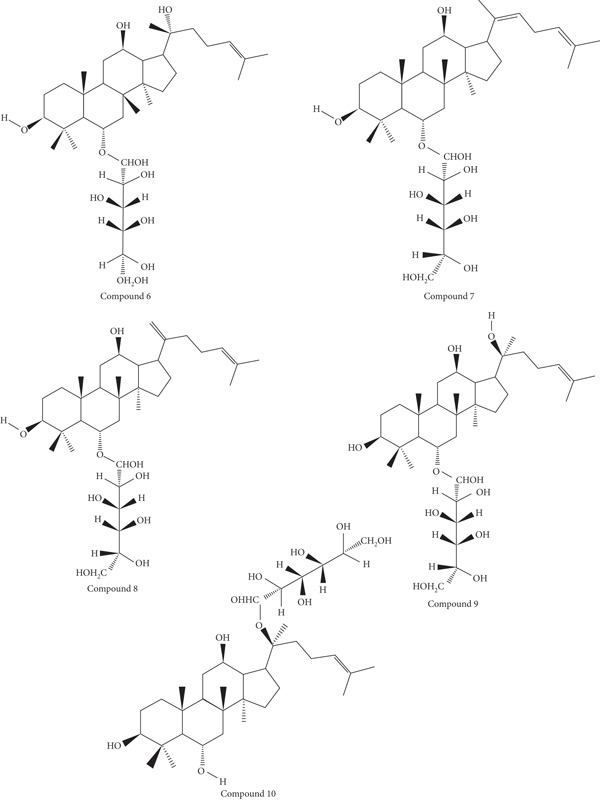
Chemical structures of ginsenosides and their derivatives selected for analysis in this study—(6) 20(R)‐protopanaxadiol (R1 = H, R2 = O − Glc), (7) Ginsenoside Rh4 (R1 = H, R2 = O − Glc), (8) Ginsenoside Rk3(R1 = H, R2 = O − Glc), (9) 20(S)‐protopanaxatriol (R1 = Glc, R2 = H), and (10) 20(S)‐protopanaxatriol (R1 = H, R2 = Glc) as reported in the literature [9]. All structures were drawn using ChemDraw software.

The molecular geometries of the compounds in Figures 1 and 2 were first constructed via the ChemDraw program, part of the ChemOffice Suite v16.0, from the available two‐dimensional representations in the literature. The two‐dimensional chemical structures were then minimized using the Chem3D module of the ChemOffice Suite v16.0. The procedure was to optimize the molecular geometry and to alleviate steric crowding, making the molecular orientations accurate. After completing the energy minimization step, the optimized molecular geometries were then transformed into the Protein Data Bank (PDB) format for greater compatibility with molecular docking software.

Follow‐up ligand optimization was performed to alter protonation states, validate bond connectivity, and determine proper charge distribution. Ligand preparation methodology was finished by using AutoDockTools (ADT) scripts integrated within the AMDock software platform. Such protocols ensured proper molecular conformations were prepared for in silico assessment, that is, docking studies against the provided protein target.

### 2.2. Preparation and Identification of Protein Target

The human placental aromatase CYP three‐dimensional crystal structure, complexed with androstenedione, was retrieved from the PDB (PDB ID: 3EQM). X‐ray diffraction methods solved the structure to a resolution of 2.90 Å and consist of a total of 503 amino acid residues [10].

**Figure 3:**
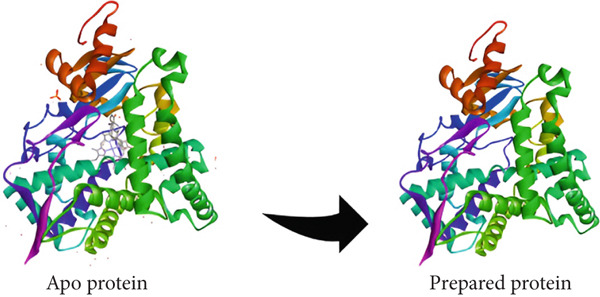
3D crystal structure of human placental aromatase cytochrome P450 in complex with androstenedione (PDB ID: 3EQM).

The resulting protein structure was properly prepared to check if it would be suitable for molecular docking analyses. Water molecules and nonrelevant heteroatoms were first stripped off to prevent unwanted interactions during docking. Hydrogen atoms were subsequently added to the protein structure to allow correct protonation states and contribute to strengthening the hydrogen bonding (H‐Bond) network (Figure 3). Residues that were missing were modeled using relevant methods to preserve the structural integrity of the protein.

The protein structure was processed and subsequently ready for docking through the utilization of ADT scripts implemented in the AMDock program. This involved assigning appropriate charges, bond order correction, and generation of a grid box around the active site to facilitate docking simulations. The prepared final ready‐to‐use protein file was stored in the format ready for use in the following molecular docking and interaction analysis with selected ligands.

### 2.3. Molecular Docking Analysis of Aromatase Enzyme With Ginsenoside and Their Derivatives

Molecular docking simulations were performed to evaluate the interactions of the human placental aromatase CYP (PDB ID: 3EQM) with some ginsenoside phytocompounds. The molecular docking experiments used the AMDock software [11], which includes the AutoDock Vina algorithm to facilitate precise and efficient docking simulations.

The AMDock method is based on the AutoDock Vina algorithm, where it identifies the most favorable binding conformation of the ligand to the target protein of interest. The approach is based on a scoring function from an empirical free energy model for predicting binding affinities. The algorithm performs a complete search of the ligand conformational space to find the best binding orientation by optimizing the free energy of the system. The goal is achieved by an iterative search mechanism that employs genetic algorithms coupled with local optimization methods to locate global energy minima.

Prior to docking, the protein was prepared by defining the active site region based on the cocrystallized ligand androstenedione. A grid box was generated around the active site to encompass all potential binding sites for the ligands. The prepared protein structure, obtained as described earlier, and the energy‐minimized ligand structures were provided as inputs to AMDock.

AMDock performs ligand and protein preparation processes automatically and correctly assigning torsions, charges, and protonation states for ligands. Docking simulations were conducted using default parameters, including flexible ligand docking, to consider the dynamic nature of ligand binding. The software positioned the ligands iteratively in the provided active site of the 3EQM protein and assessed their interaction based on binding energy.

The docking simulations led to the binding conformations that were sorted based on calculated binding affinities. The minimum binding energy of each of the ligands was considered as a reference to acquire the best ranked conformation with the most stable structural entity. The contact between the ligands and the residues found within the active site of the protein, including the hydrogen bonds, hydrophobic contacts, and van der Waals, was identified using visualizing tools such as Pymol and BIOVIA Discovery Studio Version 2021 [12].

The application of this molecular docking process facilitated the identification of potential ginsenoside compounds with high binding affinities to the aromatase enzyme, thus providing insightful information on their potential inhibitory actions and associated molecular mechanisms.

### 2.4. Molecular Dynamics (MD) Simulation

The crystal structure of human placental aromatase cytochrome P450 (PDB ID: 3EQM) in complex with androstenedione was utilized. Three specific complexes—3EQM‐COM2 (top ligand selected from docking analysis), 3EQM‐LET, and 3EQM‐ASD—were prepared for molecular dynamics simulation. Top ligands selected from docking analysis like 3EQM‐COM2, 3EQM‐LET, and 3EQM‐ASD were considered. Ligand topology was picked from the ATB server. The pdb2gmx, a GROMACS module [13], was used to add hydrogens to the heavy atoms. Prepared systems were vacuum minimized first for 1500 steps with the steepest descent algorithm. Then the structures were solvated in a cubic periodic box with a water simple point charge (SPCE) water model. The complex systems were then maintained with a proper salt concentration of 0.15 M by adding proper numbers of Na and Cl counter ions. The system preparation was mentioned based on a previously published paper [14]. Each resulting structure from the NPT equilibration phase was subjected for final production run in NPT ensemble for 200 ns simulation time. Finally, the simulation trajectory was analyzed using various tools available with the Gromacs software package, that is, the protein root mean square deviation (RMSD), root mean square fluctuation (RMSF), radius of gyration (Rg), solvent accessible surface area (SASA), and H‐Bond. Molecular Mechanics Poisson‐Boltzmann Surface Area (MM‐PBSA) method was employed to examine the binding free energy (3EQM‐COM2, 3EQM‐LET, and 3EQM‐ASD binding) of an inhibitor with protein over simulation time. A GROMACS utility g_mmpbsa was employed to estimate the binding free energy [15]. To get an accurate result, we calculated 3EQM‐COM2, 3EQM‐LET, and 3EQM‐ASD for the last 50 ns with dt 1000 frames.

### 2.5. Physicochemical Properties and Drug‐Likeness Evaluation

Physicochemical and drug‐like characteristics of the selected compounds (1–10) were investigated with SwissADME [16], an online tool used to predict the physicochemical and pharmacokinetic properties of small molecules. SwissADME examines significant molecular attributes like molecular weight (MW), partition coefficient (iLOGP), number of rotatable bonds, hydrogen acceptors and hydrogen donors, and topological polar surface area (TPSA). SwissADME also determines if the compounds are in agreement with Lipinski′s and Veber′s rules to determine if the compounds would be good drugs or not. SwissADME also gives bioavailability scores, where the ability of the molecules to get absorbed from oral intake is indicated. Use of the web server is free and does not require any log in by the user at http://www.swissadme.ch. Computer prediction data were utilized to categorize compounds based on their drug‐likeness or drug potential as drug candidates.

### 2.6. Pharmacokinetic Profiling and CYP Inhibition Prediction

Pharmacokinetic profiles of the compounds such as skin permeability (log Kp), gastrointestinal (GI) absorption, blood‐brain barrier (BBB) permeability, and Pgp substrate status were also predicted using SwissADME [16]. In addition, the inhibitory activity of the compounds towards major CYP enzymes (CYP1A2, CYP2C19, CYP2C9, CYP2D6, and CYP3A4) was also analyzed in order to assess their metabolic interactions.

### 2.7. Toxicological Assessment

The toxicity profiles of the compounds were assessed using the ProTox‐III web server [17], a robust in silico tool that combines molecular similarity and machine learning models to predict 61 endpoints of toxicity. The endpoints comprise measures of acute toxicity, organ toxicity, clinical toxicity, molecular‐initiating events (MOEs), adverse outcome (Tox21) pathways, and other toxicological factors, which also account for toxicity off‐targets. The web tool provides a broad toxicological profile comprising a confidence score for each predicted endpoint. ProTox‐III also generates a toxicity radar plot and network visualization for a thorough evaluation of toxicity. The web server is accessible freely at https://tox.charite.de without login. The 2D molecular structures of the compounds were submitted to the platform, and toxicity classification was assigned using the ProTox‐III toxicity class grading system, which ranges from Class 1 (highly toxic) to Class 6 (nontoxic), with lethal dose 50 (LD50) values in mg/kg. The classification system is useful in the assessment of potential toxicity risk of each compound.

### 2.8. Data Analysis

All the information gathered was systematically arranged and interpreted to categorize compounds based on drug‐likeness, pharmacokinetics, toxicity, and bioavailability. This categorization facilitated the selection of good drug candidates for further investigation.

## 3. Results

### 3.1. Binding Affinities of Ginsenosides and Their Derivatives With Aromatase

The molecular docking analysis with aromatase and different ginsenosides and their derivatives were performed to investigate their binding affinities, docking scores, and interaction with residues of prime importance in the active site of the enzyme. The results generated using AutoDock Vina were the binding energies, which are predictors of the relative affinity of the compounds to aromatase. A compilation of the binding affinities and corresponding parameters of ginsenosides and their derivatives with aromatase is shown in Table 1. The compounds of interest had binding affinities between −7.4 kcal/mol and −10.0 kcal/mol, of which Compound 2 has the highest at −10.0 kcal/mol, which equates to an approximate estimated *K* *i* of 46.77 nM. Compound 1 was a close second with a binding affinity of −9.9 kcal/mol and an estimated *K* *i* of 55.37 nM. The ligand efficiency (LE) values were −0.16 to −0.30, whereas the reference compound letrozole had a binding affinity of −8.4 kcal/mol with an estimated *K* *i* of 696.25 nM. The RMSD values of all the ligands were 0.000, which shows an excellent level of consistency of structural alignment throughout the docking study.

**Table 1:** Binding energies of ginsenosides and their derivatives with human placental aromatase cytochrome P450 (PDB ID: 3EQM).

**Compound**	**Binding Affinity (Kcal/mol)**	**Estimated Ki with units**	**Ligand Efficiency**	**RMSD LB**	**RMSD UB**
Compound 1	−9.9	55.37 nM	−0.29	0.000	0.000
Compound 2	−10	46.77 nM	−0.30	0.000	0.000
Compound 3	−8.8	354.46 nM	−0.27	0.000	0.000
Compound 4	−8.2	975.81 nM	−0.23	0.000	0.000
Compound 5	−7.4	3770 nM	−0.16	0.000	0.000
Compound 6	−9.0	252.91 nM	−0.20	0.000	0.000
Compound 7	−8.7	419.63 nM	−0.19	0.000	0.000
Compound 8	−8.9	299.41 nM	−0.20	0.000	0.000
Compound 9	−8.2	975.81 nM	−0.18	0.000	0.000
Compound 10	−8.8	354.46 nM	−0.19	0.000	0.000
Letrozole	−8.4	696.25 nM	−0.38	0.000	0.000

### 3.2. Molecular Docking Interaction Profile of Ginsenosides and Their Derivatives With Human Placental Aromatase CYP (PDB ID: 3EQM)

**Figure 4:**
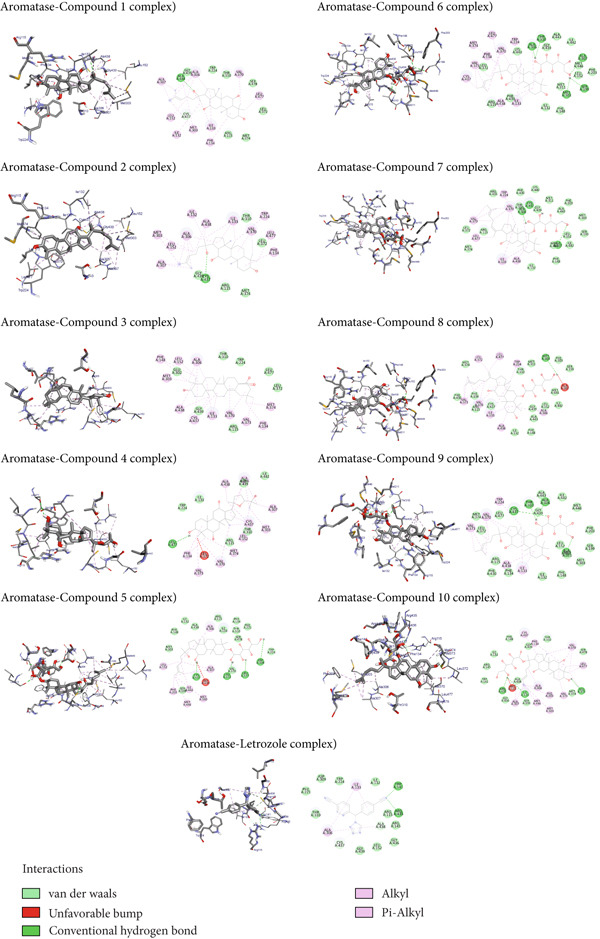
Molecular docking interaction analysis between human placental aromatase cytochrome P450 (PDB ID: 3EQM) and ginsenosides and their derivatives.

The molecular docking study of human placental aromatase CYP (PDB ID: 3EQM) with ginsenosides and their derivatives indicated significant interactions for all compounds (Figure 4). Hydrogen bonds were observed with residues such as ALA438, CYS437, and LEU477. Hydrophobic interactions involve residues like ALA306, ALA307, VAL370, LEU477, PHE134, MET303, and ILE133. Van der Waals forces were prominent with residues including GLY439, LEU372, MET374, TRP224, ARG115, and THR310. Letrozole, used as a control, exhibited interactions primarily with TRP141, ARG435, ALA306, CYS437, and THR310. These interaction profiles are critical for understanding the binding affinity and specificity of the compounds toward the aromatase enzyme.

### 3.3. MD

All‐atom MD simulation is an appropriate technique for the study of structural protein dynamics and its interaction with the ligand. It has changed the era of computer‐assisted drug discovery and design because it enables sophisticated exploration of molecular systems at the atomic level. In the present study, MD simulations were carried out in an attempt to explore the dynamic changes at the target protein binding. Some parameters like RMSD, RMSF, Rg, SASA, and inter H‐Bond were calculated for the protein and protein–ligand complex.

#### 3.3.1. RMSD

**Figure 5:**
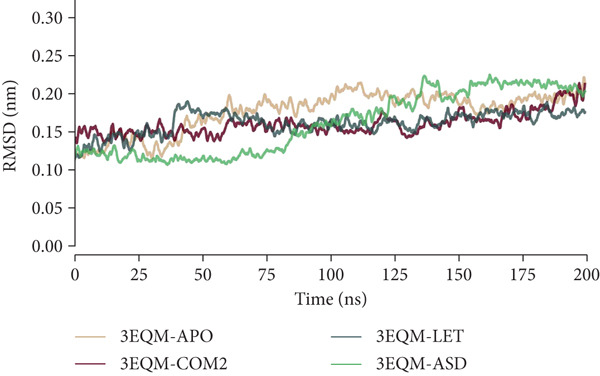
RMSD conformational dynamics analysis of 3EQM‐APO, 3EQM‐COM2, 3EQM‐LET, and 3EQM‐ASD.

To study the stability of the protein–ligand complex and to obtain information about the behavior of the systems, the RMSD values were compared for a time span (illustrated in Figure 5). The findings indicate that all systems reached equilibrium within 5 ns and had a uniform distribution through the simulated time. Also, the evaluation of the RMSD values indicated that the complexes 3EQM‐APO, 3EQM‐COM2, 3EQM‐LET, and 3EQM‐ASD were stable for 200 ns, which implies that the docked complex was stable through the simulated time. The average RMSD values were calculated to be 0.18 ± 0.03 nm for 3EQM‐APO, 0.16 ± 0.02 nm for 3EQM‐COM2, 0.16 ± 0.02 nm for 3EQM‐LET, and 0.16 ± 0.04 nm for 3EQM‐ASD. This observation implies that the systems 3EQM‐COM2, 3EQM‐LET, and 3EQM‐ASD are stable with minimal fluctuation through the simulation.

#### 3.3.2. RMSF

**Figure 6:**
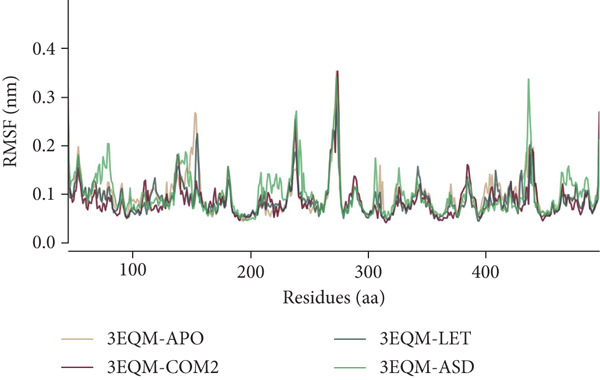
RMSF conformational dynamics analysis of 3EQM‐APO, 3EQM‐COM2, 3EQM‐LET, and 3EQM‐ASD complexes.

To compute the variations of each residue and flexible region of a protein in MD simulations, RMSF is used. By examining RMSF during simulations, the effect of ligand binding to a protein can be identified. In general, compact protein structures like sheets and helices have minimal RMSFs, and loosely ordered loop structures have larger RMSF values. In the present work, the RMSF values for each complex were computed and graphed for all residues of 3EQM‐APO, 3EQM‐COM2, 3EQM‐LET, and 3EQM‐ASD complexes (as depicted in Figure 6). The average values of RMSF of 3EQM‐APO, 3EQM‐COM2, 3EQM‐LET, and 3EQM‐ASD were 0.10 ± 0.04 nm, 0.09 ± 0.04 nm, 0.09 ± 0.03 nm, and 0.10 ± 0.04 nm, respectively. The findings show that the 3EQM‐APO, 3EQM‐COM2, 3EQM‐LET, and 3EQM‐ASD complex was not capable of altering the overall RMSF distribution effectively.

#### 3.3.3. RG

**Figure 7:**
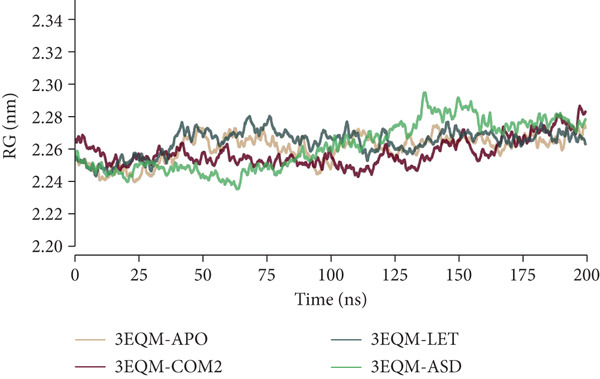
Rg conformational dynamics analysis of 3EQM‐APO, 3EQM‐COM2, 3EQM‐LET, and 3EQM‐ASD complexes.

To contrast the dynamic compactness and stability of the 3EQM‐APO, 3EQM‐COM2, 3EQM‐LET, and 3EQM‐ASD complex structures, Rg values were computed and plotted graphically against time, as shown in Figure 7. The average Rg values of the 3EQM‐APO complex, 3EQM‐COM2 complex, 3EQM‐LET complex, and 3EQM‐ASD complex were 2.26 ± 0.01 nm, 2.26 ± 0.01 nm, 2.26 ± 0.01 nm, and 2.26 ± 0.02 nm, respectively. The 3EQM‐COM2 and 3EQM‐LET complex systems possessed Rg values that were identical to those of the 3EQM‐ASD complex system, and hence, the compactness of the two complex systems was identical.

#### 3.3.4. SASA

**Figure 8:**
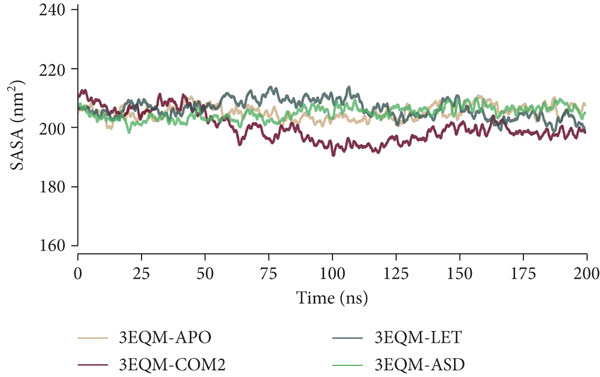
SASA conformational dynamics analysis of 3EQM‐APO, 3EQM‐COM2, 3EQM‐LET, and 3EQM‐ASD complexes.

To estimate the solvent accessibility of a protein molecule in a solvent environment, SASA is a significant parameter. SASA values were calculated and graphically plotted in this research to estimate the effect of 3EQM‐ASD binding on the solvent accessibility of the target (see Figure 8). The graph shows the same trend of SASA values for 3EQM‐APO, 3EQM‐COM2, 3EQM‐LET, and 3EQM‐ASD. The average SASA values of 3EQM‐APO, 3EQM‐COM2, 3EQM‐LET, and 3EQM‐ASD were 205.35 ± 3.06 nm, 200.34 ± 5.37 nm, 206.57 ± 3.73 nm, and 205.18 ± 2.99 nm, respectively. The SASA values reveal a good equilibrium with minor fluctuations during the simulation.

#### 3.3.5. Intra‐Hydrogen and Inter‐Hydrogen Bond

**Figure 9:**
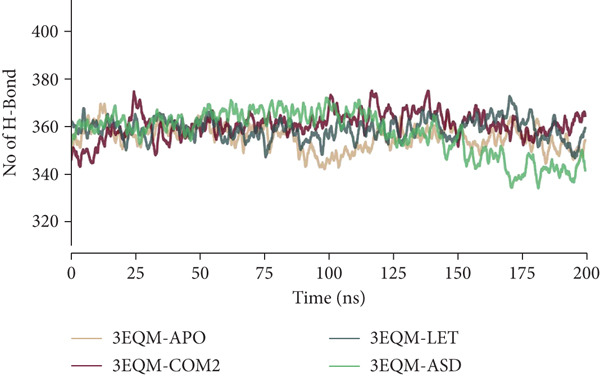
Intramolecular hydrogen bonds of 3EQM‐APO, 3EQM‐COM2, 3EQM‐LET, and 3EQM‐ASD during the simulation time.

To determine the stability of protein alone (3EQM‐APO, 3EQM‐COM2, 3EQM‐LET, and 3EQM‐ASD complexes.) interactions, the intra‐hydrogen and inter‐hydrogen bond formation plays a significant role. In this research, we examined the time‐dependent behavior of intra‐hydrogen bonds 3EQM‐APO, 3EQM‐COM2, 3EQM‐LET, and 3EQM‐ASD complexes and graphed the results (Figure 9). The average intra–H‐bond values for 3EQM‐APO, 3EQM‐COM2, 3EQM‐LET, and 3EQM‐ASD complexes were found to be 355.52 ± 9.38 nm, 360.77 ± 9.69 nm, 358.98 ± 9.14 nm, and 357.62 ± 12.18 nm, respectively. The plot showed that even though higher hydrogen bonds were formed in the 3EQM‐COM2, 3EQM‐LET, and 3EQM‐ASD complex than in the 3EQM‐APO form of the protein, the result showed that, 3EQM‐COM2, 3EQM‐LET, and 3EQM‐ASD complex was more stable than the 3EQM‐APO form.

**Figure 10:**
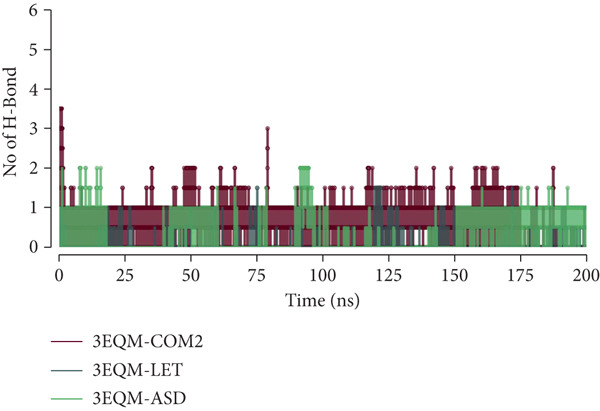
Intermolecular hydrogen bonds between protein–ligand during the simulation time.

In protein–ligand interaction stability, the formation of hydrogen bonds is of utmost importance. The present work concerned the time‐dependent behavior of hydrogen bonds of 3EQM‐APO, 3EQM‐COM2, 3EQM‐LET, and 3EQM‐ASD, and the findings are illustrated in Figure 10. Based on our findings, we observed that the docked complex remained stable throughout the simulation, backed by a minimum of 1–6 hydrogen bonds with the 3EQM‐COM2 and 3EQM‐LET complexes, and 1–4 hydrogen bonds with the 3EQM‐ASD complex.

#### 3.3.6. Principal Component Analysis (PCA)

To explore the collective motions in 3EQM‐APO, 3EQM‐COM2, 3EQM‐LET, and 3EQM‐ASD, PCA was carried out. The first eigenvectors (EVs) play a central role in identifying the global motion of a protein molecule. Accordingly, PCA was carried out to study the conformational dynamics of 3EQM‐APO, 3EQM‐COM2, 3EQM‐LET, and 3EQM‐ASD along the simulation (Figure 11). The time evolution of PCA shows that the overall flexibility of the complex 3EQM‐APO, 3EQM‐COM2, 3EQM‐LET, and 3EQM‐ASD reduced along both EVs, suggesting enhanced stability (Figure 11). The graphical representation clearly indicates that the complex 3EQM‐APO, 3EQM‐COM2, 3EQM‐LET, and 3EQM‐ASD spanned nearly all conformational motions and suggested overlap. Overall, the lowered level of movements observed in 3EQM‐LET and 3EQM‐ASD suggests that these mutants had no vital effect on the target conformation and dynamics, thus enhancing the stability of the complex.

Figure 11Principal component analysis 2D projection plot shows the conformation sampling of 3EQM‐APO, 3EQM‐COM2, 3EQM‐LET, and 3EQM‐ASD on PC1 and PC2.

**Figure:**
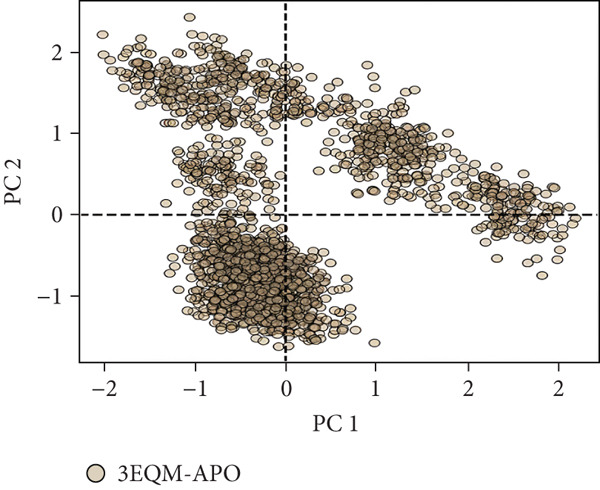
(a)

**Figure:**
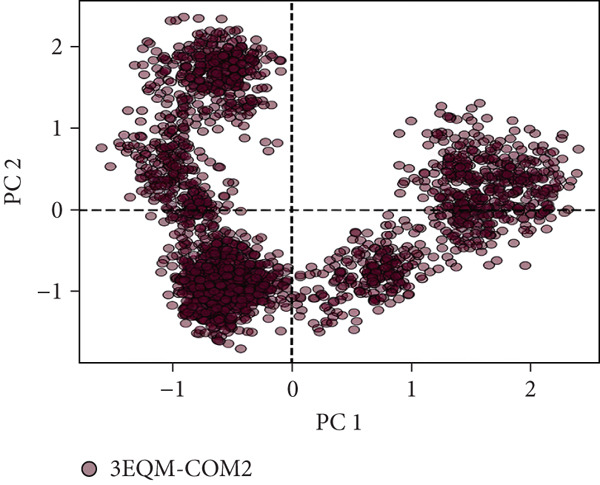
(b)

**Figure:**
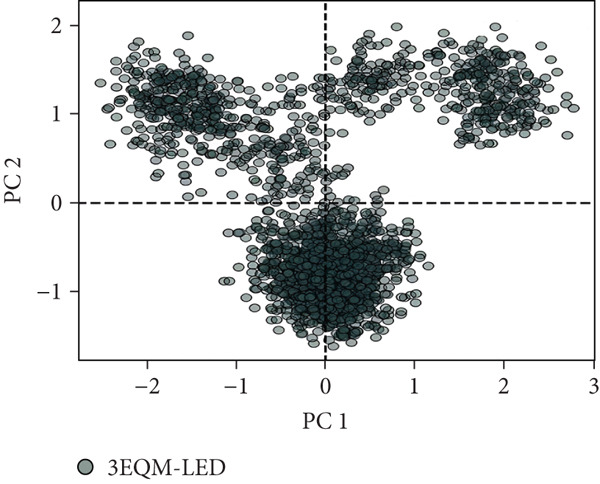
(c)

**Figure:**
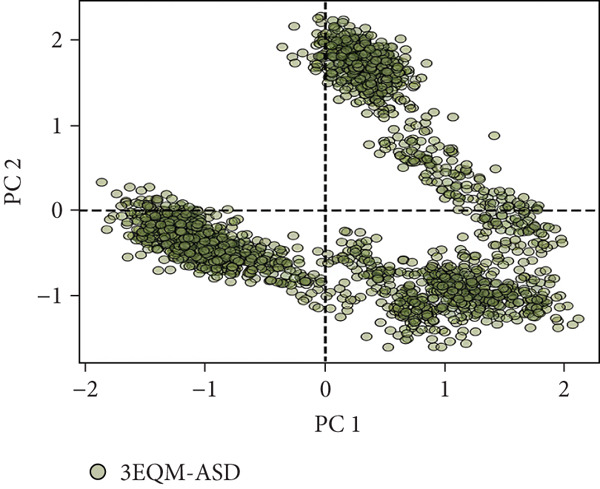
(d)

#### 3.3.7. Free Energy Landscapes (FELs)

FEL plot is a common approach for the study of the mechanism of protein folding and global stability. FEL plots are a graphical representation of the most stable ensembles of conformations of a given protein structure. In this work, we built FEL plots for PC1 and PC2 (Figure 12), where areas filled with dark blue color correspond to a more stable protein conformation of lower energy. The plots present energy values from 0 to 14 kJ/mol for 3EQM‐APO and from 0 to 16 kJ/mol for 3EQM‐COM2, 3EQM‐LET, and 3EQM‐ASD simulations, respectively. The FEL plots show that the 3EQM‐LET and 3EQM‐ASD complexes have one global minimum, which is located in a deep local well. These findings suggest that 3EQM‐LET and 3EQM‐ASD do not exert any significant conformational effect on the target structure and thus stabilize it (Figure 12).

Figure 12The free energy landscape plots for (a) 3EQM‐APO, (b) 3EQM‐COM2, (c)3EQM‐LET, (d) and (e) 3EQM‐ASD complexes.

**Figure:**
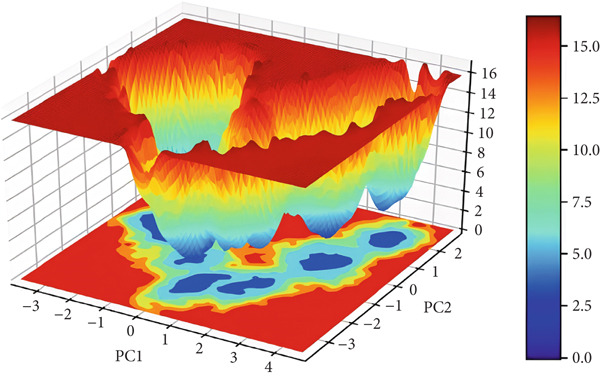
(a)

**Figure:**
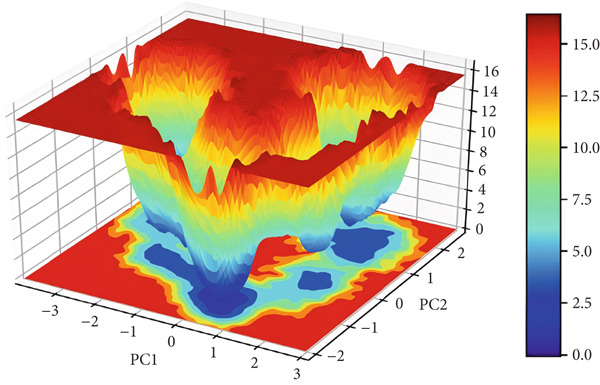
(b)

**Figure:**
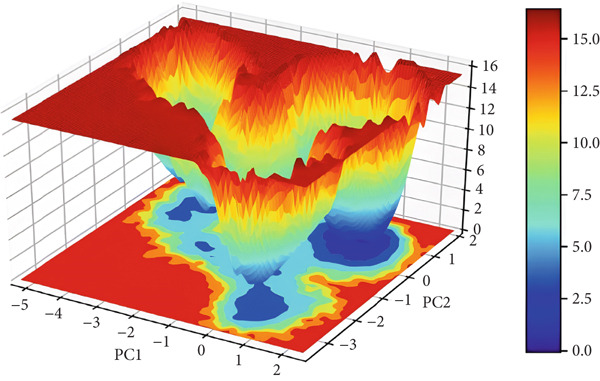
(c)

**Figure:**
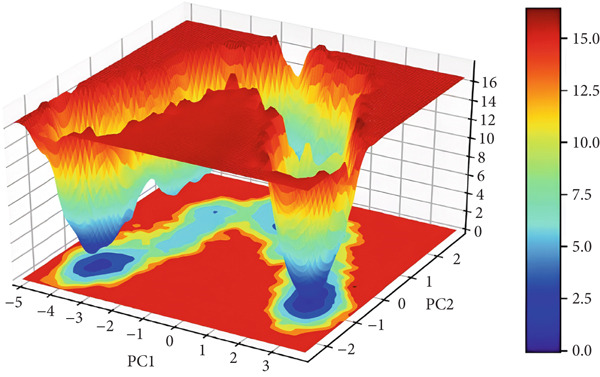
(d)

#### 3.3.8. MM‐PBSA

To find the binding affinity of 3EQM‐COM2, 3EQM‐LET, and 3EQM‐ASD, we analyzed the relative binding strength in the protein from the perspective of summary energy. Table 2 presents the comparison of binding strength of 3EQM‐COM2, 3EQM‐LET, and 3EQM‐ASD with inhibitors calculated by the MM‐PBSA method. Along a common simulation trajectory, we compute the contribution of individual residues to the interaction energy.

**Table 2:** Binding strength of protein–ligand complexes via the MM‐PBSA method.

**System**	**Van der Waal energy**	**Electrostatic energy**	**Polar solvation energy**	**Binding energy**
3EQM‐COM2	−237.217 +/−12.741 kJ/mol	−28.360 +/−6.797 kJ/mol	166.736 +/−23.018 kJ/mol	−123.479 +/−18.967 kJ/mol
3EQM‐LET	−140.290 +/−8.426 kJ/mol	−12.382 +/−6.834 kJ/mol	98.136 +/−8.176 kJ/mol	−70.448 +/−6.275 kJ/mol
3EQM‐ASD	−142.408 +/−9.692 kJ/mol	−28.963 +/−4.457 kJ/mol	69.741 +/−4.870 kJ/mol	−117.214 +/−9.870 kJ/mol

### 3.4. Physicochemical Properties and Drug‐Likeness Assessment of Ginsenosides and Their Derivatives and Letrozole

The physicochemical properties and drug‐likeness of ginseng derivatives (Compounds 1–10) and letrozole were evaluated (Table 3). The MWs of the ginseng derivatives ranged from 456.7 g/mol to 656.89 g/mol. Notably, Compounds 5–10 exceeded the 500 g/mol threshold, which can present challenges for membrane permeability and oral bioavailability.

**Table 3:** Molecular properties and drug‐likeness evaluation of ginsenosides and their derivatives and letrozole.

**Compound Name**	**MW**	**ILOGP**	**Rotatable bonds**	**H-bond acceptors**	**H-bond donors**	**TPSA**	**Lipinski violations**	**Veber violations**	**Bioavailability Score**
Compound 1	476.73	4.42	4	4	4	80.92	1	0	0.55
Compound 2	460.73	5.13	4	3	3	60.69	1	0	0.55
Compound 3	456.7	3.89	1	3	2	57.53	1	0	0.85
Compound 4	492.73	4.07	2	5	4	90.15	0	0	0.55
Compound 5	640.89	2.51	11	9	8	171.07	2	2	0.17
Compound 6	656.89	3.53	11	10	9	191.3	2	2	0.17
Compound 7	638.87	4.76	10	9	8	171.07	2	1	0.17
Compound 8	638.87	3.17	11	9	8	171.07	2	2	0.17
Compound 9	656.89	3.67	11	10	9	191.3	2	2	0.17
Compound 10	656.89	4.18	11	10	9	191.3	2	2	0.17
Letrozole	285.3	2.2	3	4	0	78.29	0	0	0.55

Further analysis revealed that Compounds 5–10 also violated Lipinski′s and Veber′s rules, primarily due to their high MW and large number of rotatable bonds and hydrogen bond acceptors/donors. These violations are strong indicators of potential issues with oral absorption, a prediction supported by their very low calculated bioavailability score of 0.17. In contrast, Compounds 1–4 showed greater compliance with these rules, suggesting a more favorable profile for development as oral therapeutics. Particularly noteworthy is Compound 3, which achieved the highest predicted bioavailability score of all tested compounds (0.85). Letrozole, an established oral drug, adhered to all rules with a MW of 285.3 g/mol, a TPSA of 78.29 Å^2^, and a favorable bioavailability score of 0.55, serving as a benchmark for desirable drug‐like properties.

### 3.5. Pharmacokinetic and CYP Inhibition Profiles of Ginsenosides and Their Derivatives and Letrozole

The pharmacokinetic and metabolic profiles of ginseng derivatives (Compounds 1–10) and letrozole were determined (Table 4). A critical parameter for oral drug viability, GI absorption, was predicted to be high for Compounds 1, 2, and 4, positioning them as promising candidates for oral administration.

**Table 4:** Pharmacokinetic properties and CYP enzyme inhibition profiles of ginsenosides and their derivatives and letrozole.

**Compound Name**	**Log Kp (cm/s)**	**GI absorption**	**BBB permanent**	**PGP substrate**	**CYP1A2 inhibitor**	**CYP2C19 inhibitor**	**CYP2C9 inhibitor**	**CYP2D6 inhibitor**	**CYP3A4 inhibitor**
Compound 1	−5.05	High	No	Yes	No	No	No	No	No
Compound 2	−4	High	No	No	No	No	No	No	No
Compound 3	−3.77	Low	No	No	No	No	No	No	No
Compound 4	−6.07	High	No	Yes	No	No	No	No	No
Compound 5	−7.33	Low	No	Yes	No	No	No	No	Yes
Compound 6	−8.38	Low	No	Yes	No	No	No	No	No
Compound 7	−7.31	Low	No	Yes	No	No	No	No	No
Compound 8	−7.26	Low	No	Yes	No	No	No	No	No
Compound 9	−8.38	Low	No	Yes	No	No	No	No	No
Compound 10	−8.38	Low	No	Yes	No	No	No	No	No
Letrozole	−6.1	High	Yes	No	Yes	Yes	Yes	Yes	No

**Figure 13:**
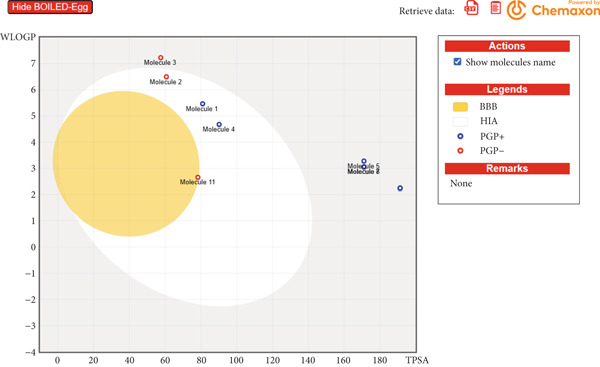
BOILED‐Egg model analysis of ginsenosides and their derivatives and letrozole.

Importantly, the prediction that none of the ginseng derivatives were permeable to the BBB was consistent across different outputs from the SwissADME tool. As explicitly stated in Table 4 (“BBB permeant: No”) and visually confirmed by the BOILED‐Egg plot (Figure 13), where all ginseng derivatives lie outside the BBB‐permeation space, these compounds are not expected to enter the central nervous system. This is a highly desirable safety feature for an aromatase inhibitor, as it minimizes the potential for CNS‐related side effects. Letrozole, by contrast, was consistently predicted to be BBB permeant. Furthermore, most ginseng derivatives were identified as substrates of P‐glycoprotein (PGP), an efflux pump that actively removes compounds from the brain, which further supports their low potential for CNS entry. In terms of metabolic stability, only Compound 5 was predicted to inhibit CYP3A4, whereas letrozole showed broad inhibitory activity across multiple CYP enzymes, suggesting the ginseng derivatives may have a lower risk of drug–drug interactions.

### 3.6. Comparative Toxicity Study of Ginsenosides and Their Derivatives and Letrozole

The toxicity profiles were evaluated based on predicted LD50 values and specific toxicological endpoints (Table 5). The LD50 values for the ginseng derivatives ranged from 1700 mg/kg to 8000 mg/kg, corresponding to toxicity Classes 4–6. These classifications (where Class 4 is “harmful if swallowed” and Class 6 is “nontoxic”) indicate a generally low order of acute toxicity, which is a favorable preliminary safety profile for a drug candidate.

**Table 5:** Toxicity profiling of ginsenosides and their derivatives and letrozole.

**Compound Name**	**LD50 Value**	**Toxicity Class**	**Hepatotoxicity**	**Carcinogenicity**	**Immunotoxicity**	**Mutagenicity**	**Cytotoxicity**
Compound 1	2260 mg/kg	5	Inactive	Active	Active	Inactive	Inactive
Compound 2	2260 mg/kg	5	Inactive	Active	Active	Inactive	Inactive
Compound 3	2000 mg/kg	4	Active	Active	Active	Inactive	Inactive
Compound 4	2280 mg/kg	5	Inactive	Inactive	Active	Inactive	Inactive
Compound 5	4000 mg/kg	5	Inactive	Inactive	Active	Inactive	Inactive
Compound 6	4000 mg/kg	5	Inactive	Inactive	Active	Inactive	Inactive
Compound 7	1700 mg/kg	4	Inactive	Inactive	Active	Inactive	Inactive
Compound 8	8000 mg/kg	6	Inactive	Inactive	Active	Inactive	Inactive
Compound 9	4000 mg/kg	5	Inactive	Inactive	Active	Inactive	Inactive
Compound 10	5190 mg/kg	6	Inactive	Inactive	Active	Inactive	Inactive
Letrozole	1463 mg/kg	4	Inactive	Active	Inactive	Inactive	Inactive

Compound 8 was identified as the safest derivative in silico, with the highest LD50 value (8000 mg/kg) and a nontoxic Class 6 rating. Most derivatives were predicted to be inactive for hepatotoxicity, mutagenicity, and cytotoxicity. However, the prediction of active carcinogenicity for Compounds 1, 2, and 3, similar to the reference drug letrozole, highlights a potential area of concern that would require careful experimental validation in preclinical safety studies. The predicted immunotoxicity for most derivatives also warrants further investigation.

### 3.7. BOILED‐Egg Model Assessment of Ginsenosides and Their Derivatives and Letrozole

The BOILED‐Egg model (Figure 13) was used to predict the passive human intestinal absorption (HIA) and BBB permeability of the ginseng derivatives and the reference drug, letrozole. The model′s white region represents the optimal physicochemical space for high HIA, whereas the yellow yolk region indicates a high probability of BBB penetration.

The analysis reveals that all tested compounds (Molecules 1–10) fall within the white region, predicting that all of the ginseng derivatives, as well as letrozole, are likely to be well‐absorbed by the GI tract.

Crucially, a clear distinction in BBB permeability was observed. All of the evaluated ginseng derivatives (represented by Molecules 1 through 10 in the plot) are located outside the yellow yolk region. This predicts that these compounds will not cross the BBB. In contrast, the control drug, Molecule 11 (letrozole), is located inside the yellow yolk, indicating a high probability of BBB penetration.

This finding is significant for drug development, as the therapeutic target (aromatase) for breast cancer is primarily in peripheral tissues. The predicted inability of the ginseng derivatives to enter the central nervous system suggests a potentially safer profile with a lower risk of CNS‐related side effects compared to BBB‐penetrant inhibitors.

Furthermore, the model distinguishes between substrates for the PGP efflux pump. Molecules 2, 3, and 11 are predicted to be nonsubstrates (PGP−), whereas the other compounds are predicted to be substrates (PGP+). The PGP− status of letrozole further supports its potential to accumulate in the brain, as it would not be actively removed by this key efflux transporter.

## 4. Discussion

In the present study, a critical assessment was performed on the binding potential, physicochemical characteristics, pharmacokinetic profiles, and toxicity of various ginsenosides and their derivatives as promising aromatase inhibitors. Aromatase is a crucial enzyme in estrogen biosynthesis, and its inhibition is a crucial part of the treatment protocol for hormone‐dependent cancers, in particular, breast cancer [18]. Our results identified Compound 2 to be the most promising candidate, with the highest binding affinity of −10.0 kcal/mol, better LE, and good pharmacokinetic behavior. The results suggest that Compound 2 is a highly promising candidate as an anticancer drug for hormone‐dependent cancers. Molecular docking analysis revealed that Compound 2 interacted excellently with crucial active site residues of aromatase like ALA438, CYS437, and LEU477, which are vital for the inhibition of the enzyme. The estimated *K* *i* value of 46.77 nM calculated further validated its enormous binding potential. Contrarily, letrozole, a highly effective aromatase inhibitor, exhibited interactions with residues such as TRP141, ARG435, and ALA306, revealing the differences in the binding mechanisms and potential benefits associated with ginsenosides and their derivatives. The findings are consistent with earlier results that underscore the importance of targeting specific active site residues to increase the potency of the inhibitor (structure–activity relationship and in silico docking analysis of dicarboximide fungicides on 17*β*‐hydroxysteroid dehydrogenase 1 of human, rat, and pig 2025) [19].

Comparing our results with existing research, it is clear that ginsenosides and their derivatives exhibit binding affinities similar to or even better than those of traditional inhibitors such as letrozole. For example, a research study on okra‐derived compounds as aromatase inhibitors identified quercetin 3‐gentiobioside as an outstanding candidate. This compound had a binding affinity of −10 kcal/mol, which is higher than that of letrozole (−8.2 kcal/mol) [3]. Likewise, *Centella asiatica* compounds were found to have binding affinities of −8.4 kcal/mol, further validating the use of phytocompounds as good aromatase inhibitors [20]. However, such findings point to the potential of phytocompounds as good aromatase inhibitors for hormone‐dependent cancers. Protopanaxadiol′s −10.0 kcal/mol binding affinity puts it at the top of the list of potential phytocompound‐based inhibitors. Depending on the derivatives, however, higher MWs (e.g., 456.7–656.89 g/mol) may pose drug delivery challenges compared with the MW of letrozole (285.3 g/mol). Such challenges are consistent with existing observations that MW is inversely related to oral bioavailability and overall drug‐likeness [21]. Additionally, Compounds 5–10, which break Lipinski′s and Veber′s rules, can be expected to undergo structural optimization to improve bioavailability without sacrificing efficacy. Notwithstanding such compromises, our research proved that Compounds 1, 2, and 4 had high GI absorption, thus making them good candidates for oral administration. In contrast to some research studies that only consider binding affinity, our incorporation of MD simulations added insight into the stability of protein–ligand complexes over 200 ns, showing similar stability between 3EQM‐COM2 and 3EQM‐LET complexes. The methodological approach taken in this research focuses on the robustness of our findings while, at the same time, overcoming the shortcomings of earlier studies that did not have such stringent validation [22]. Interestingly, none of the derivatives demonstrated significant BBB permeability, which, although limiting their application for central nervous system disorders, enhances their specificity and reduces the risk of off‐target effects and associated toxicities.

The main hypothesis of this study postulated that ginsenosides and their derivatives could be effective aromatase inhibitors, and the findings justify this contention, especially for Compound 2 (protopanaxadiol). This study highlights the potential of phytocompound‐derived aromatase inhibitors to address the demand for novel treatments for hormone‐dependent cancers. Secondly, the determination of pharmacokinetic profiles and toxicology parameters provides an adequate platform for guiding these compounds to preclinical and clinical trials. However, some of the issues left unanswered are the long‐term safety of these derivatives and their effectiveness in causing apoptosis in breast cancer cells. Future studies must be directed towards in vitro and in vivo validations in order to determine the therapeutic potential of Protopanaxadiol, as well as structural modifications to improve its pharmacokinetic characteristics. In addition, the study of combination therapy with existing inhibitors like letrozole might result in synergistic effects, thus enhancing therapeutic efficacy and potentially reducing the risk of resistance development. Since this study bridges computational prediction and experimental feasibility, the synergy of these two approaches will be the platform for the clinical use of ginsenosides and their derivatives as aromatase inhibitors.

## 5. Conclusion

This study elucidates the molecular interactions behind the activity of ginsenosides and their derivatives as aromatase inhibitors, and identifies their potential in the treatment of hormone‐dependent cancers. Notably, the protopanaxadiol‐aromatase complex had very restricted conformational mobility, hence confirming its potential as a potent and stable inhibitor. Further, its good pharmacokinetics and low toxicity confirm its high potential as a therapeutic molecule. These findings provide a solid foundation for advancing protopanaxadiol into preclinical and clinical trials to evaluate its safety and efficacy. The insight gained also has the potential to guide the design of new analogs or derivatives to enhance the treatment of hormone‐dependent breast cancer. These molecular interaction insights support the development of phytocompound‐based therapies, alone or with conventional treatments.

## Conflicts of Interest

The authors declare no conflicts of interest.

## Author Contributions

Jayasri Gokila Madhan: Data collection and data analysis. Surya Sekaran: Data collection, data analysis and data Interpretation, drafting the article, and critical revision of the article. Rajeswari Nambirajan Akshaya: Data collection and data analysis. Khavyanjali Venkatesan: Data collection and data analysis. Kavin Kirupanandasamy: Drafting the article. Kiruthika Vijayakumar: Drafting the article and critical revision of the article. Nancy Jenifer: Drafting the article, and critical revision of the article. Benedict Christopher Paul: Conception of the work, critical revision of the article, and final approval of the version to be published. Jayasri Gokila Madhan and Surya Sekaran share the first authorship.

## Funding

No funding was received for this manuscript.

## Data Availability

The authors confirm that the data supporting the findings of this study are available within the article.
